# Supplementary material for: Accurate cross-species 5mC detection for Oxford Nanopore sequencing in plants with DeepPlant
Source: Nat Commun. 2025 Apr 4;16:3227. doi: 10.1038/s41467-025-58576-x (PMC11971355; doi:10.1038/s41467-025-58576-x)
Supplement: Supplementary file 1 — Supplementary Information [file 41467_2025_58576_MOESM1_ESM.pdf]

Accurate cross-species 5mC detection for Oxford Nanopore sequencing in plants with DeepPlant

Chen *et al.*

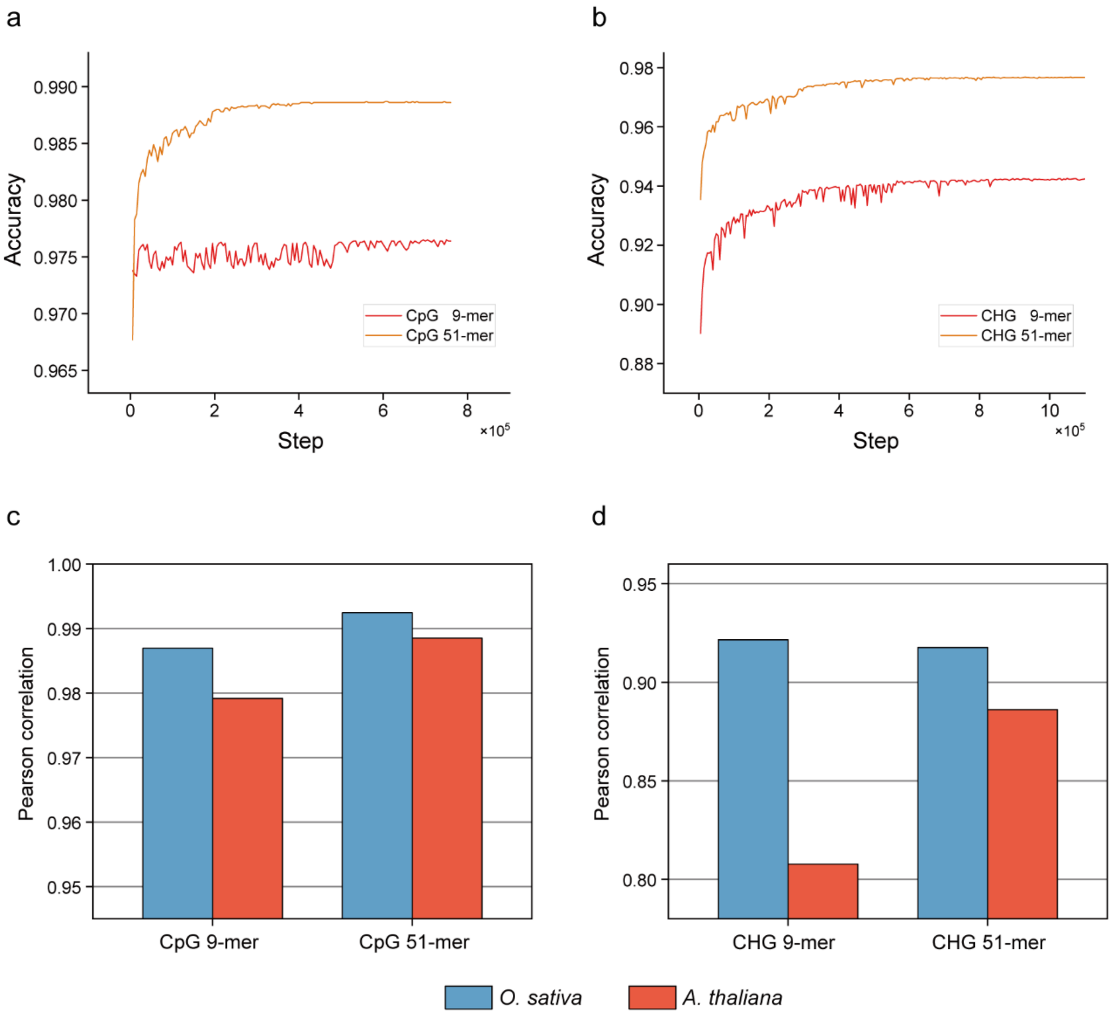

Supplementary Fig. 1. Training accuracy and methylation frequency evaluation for CpG and CHG methylation detection. a, b, Accuracy progression during training for CpG and CHG methylation detection, respectively. **c, d,** Methylation quantitative evaluations for CpG and CHG methylation. Single chromosome methylation frequency correlations with corresponding BS-seq data in *A. thaliana* and *O. sativa* are shown in panels **c**,**d**. Source data are provided as a Source Data file.

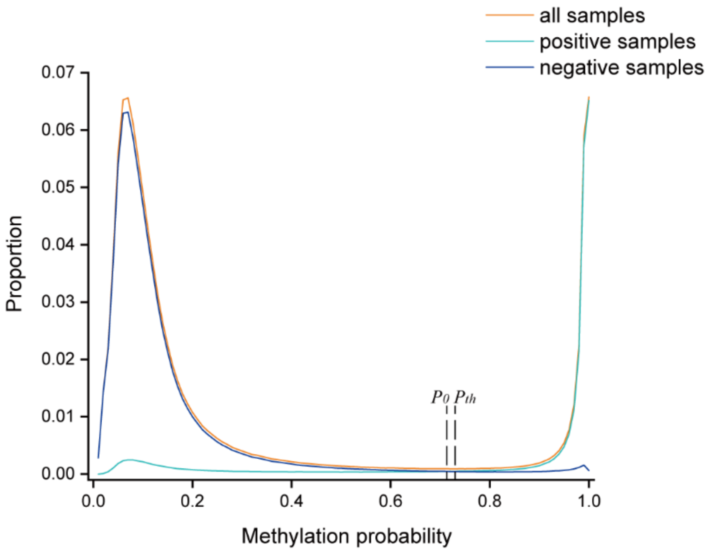

Supplementary Fig. 2. Threshold selection method for site-level evaluation in DeepPlant. The figure shows the distribution of model's output probabilities for a large number of positive samples (BS-seq with 100% methylation frequency) and negative samples (BS-seq with 0% methylation frequency) from *S. lycopersicum*, along with the probability distribution for all of these samples. The ratio of positive to negative samples is 1:3. To facilitate the explanation of the method, only high-confidence samples were selected in the figure. The vertical axis represents the proportion of sample count to the all samples count (including both positive and negative). The intersection point of the probability distribution curves for positive and negative samples corresponds to the probability value P_0, which serves as the optimal methylation threshold. This threshold balances the model's false positives and false negatives, minimizing their total (the sum of the area under the negative curve to the right of this point and the area under the positive curve to the left of this point will reach its minimum value). The lowest point of the total sample probability distribution curve corresponds to the probability value P_{th}, which is near P_0. P_{th} can be approximated as the optimal methylation threshold. Source data are provided as a Source Data file.

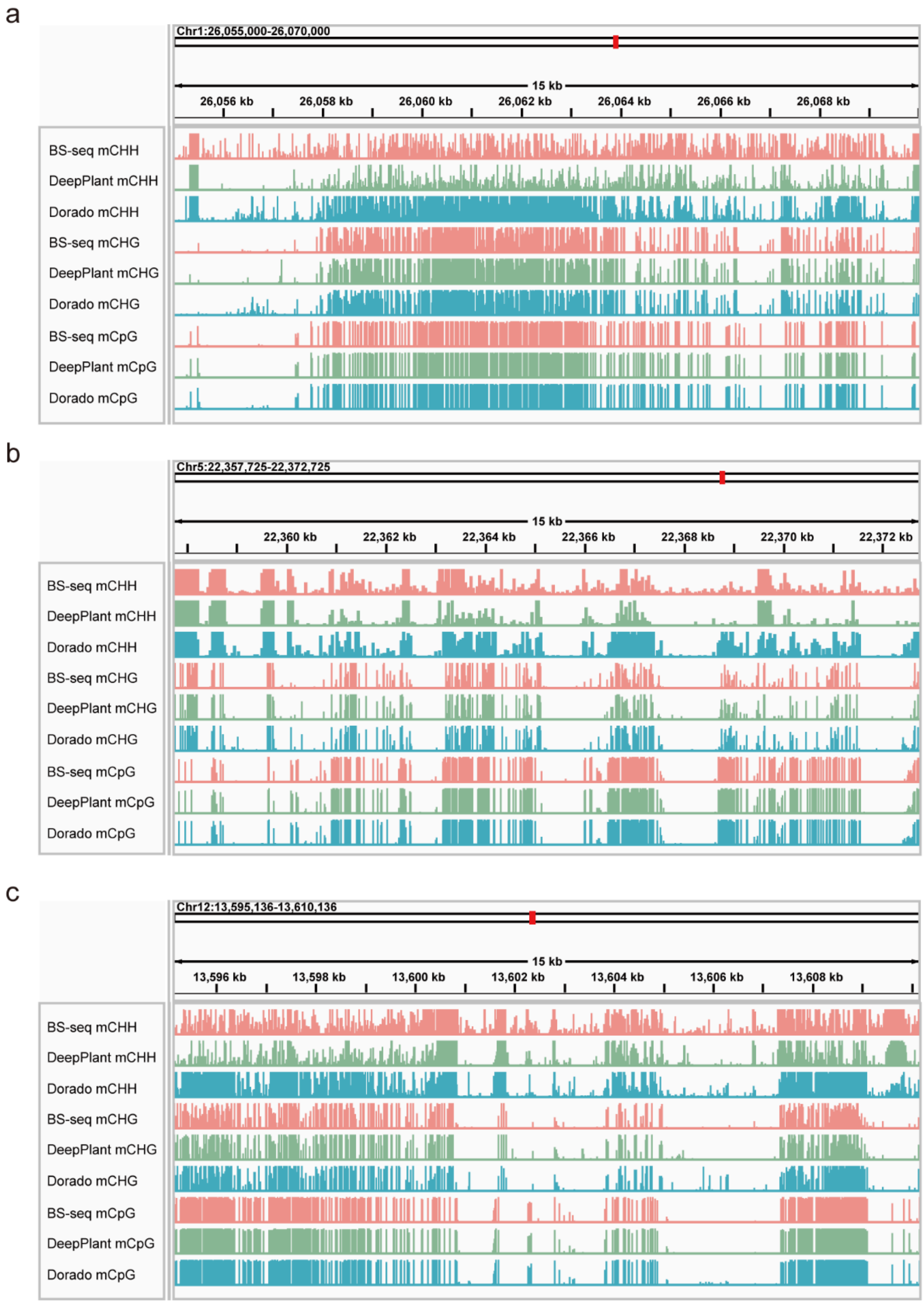

Supplementary Fig. 3. CHH, CHG, and CpG methylation analyses in regions of comprehensive coverage by both BS-seq and nanopore sequencing in *Oryza sativa*. a, b, c, Genome browser view showing CHH, CHG and CpG methylation status in the comprehensive coverage regions of Chr1, Chr5 and Chr12. The graphs showed profiled methylation frequencies on CHHs, CHGs and CpGs with at least 10 reads coverage. Source data are provided in Zenodo [https://doi.org/10.5281/zenodo.15062213].

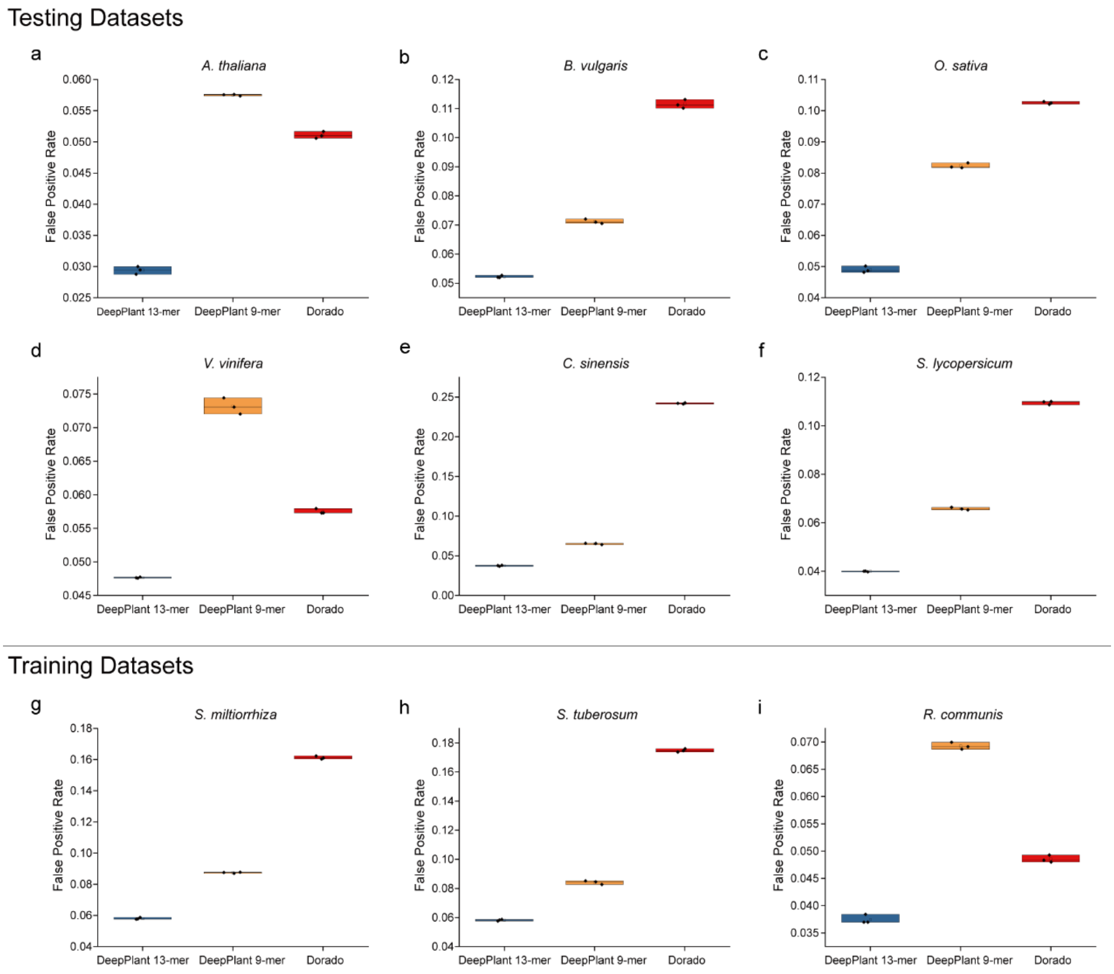

Supplementary Fig. 4. False positive rates of DeepPlant and Dorado for CHH methylation detection across nine species at the single-molecule level. a-f, false positive rates of single-molecule level evaluations across the *A. thaliana* (**a**), *B. vulgaris* (**b**), *O. sativa* (**c**), *V. vinifera* (**d**), *C. sinensis* (**e**), and *S. lycopersicum* (**f**) datasets. **g-i,** false positive rates across the three training datasets, *S. miltiorrhiza* (**g**), *S. tuberosum* (**h**), and *R. communis* (**i**). The center line represents the median; each box shows the first and third quartiles; solid dots represent data, and hollow dots represent the average value. Source data are provided as a Source Data file.

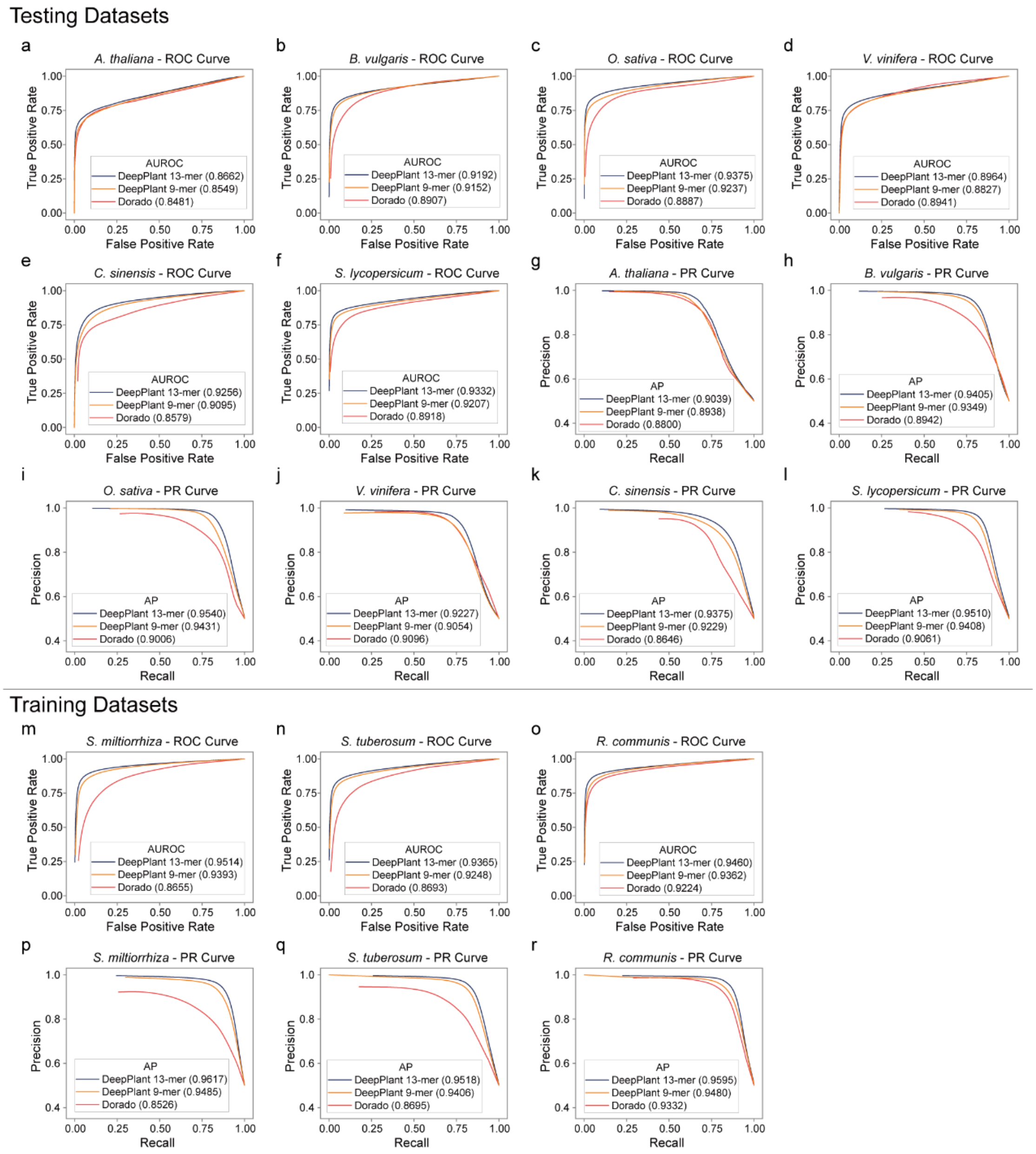

Supplementary Fig. 5. Receiver operating characteristic (ROC) curves and precision-recall (PR) curves for DeepPlant and Dorado across nine datasets. a-f, ROC curves of single-molecule level evaluations across the *A. thaliana* (**a**), *B. vulgaris* (**b**), *O. sativa* (**c**), *V. vinifera* (**d**), *C. sinensis* (**e**), and *S. lycopersicum* (**f**) datasets. **g-l,** PR curves single-molecule level evaluations across the *A. thaliana* (**g**), *B. vulgaris* (**h**), *O. sativa* (**i**), *V. vinifera* (**j**), *C. sinensis* (**k**), and *S. lycopersicum* (**l**) datasets. **m-o,** ROC curves across the three training datasets, *S. miltiorrhiza* (**m**), *S. tuberosum* (**n**), and *R. communis* (**o**). **p-r,** PR curves across the training datasets, *S. miltiorrhiza* (**p**), *S. tuberosum* (**q**), and *R. communis* (**r**). AUROC, area under ROC; AP, area under PR curve. Source data are provided as a Source Data file.

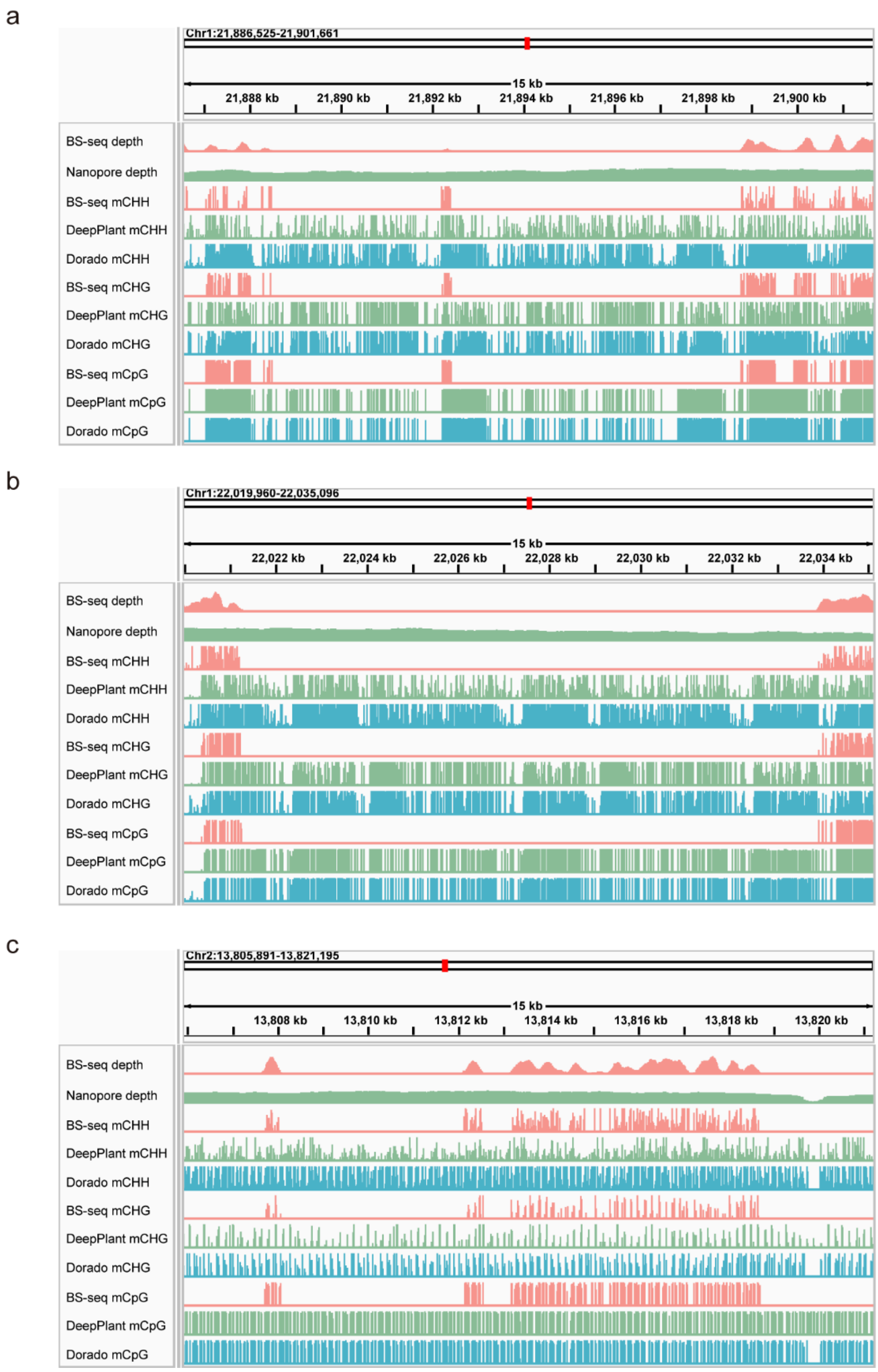

Supplementary Fig. 6. CHH, CHG and CpG methylation analyses in centromeric and transposable element regions of *Oryza sativa*. a, b, c, Genome browser view showing read coverage and CHH, CHG and CpG methylation status in the centromeric regions of Chr1 and Chr2, comparing whole-genome BS-seq and nanopore data (analyzed by DeepPlant and Dorado). In each graph, the top two panels showed normalized read coverage for BS-seq and nanopore, respectively; and the bottom panels showed profiled methylation frequencies on CHHs, CHGs and CpGs with at least 10 reads coverage. Source data are provided in Zenodo [https://doi.org/10.5281/zenodo.15062213].

Supplementary Note 1. Quantitative performance evaluation of DeepPlant for CpG and CHG methylation detection

In genome-wide evaluations for CHG methylation, DeepPlant slightly outperformed Dorado across nine species. At lower sequencing depths (≤10×), the performance difference remained within 1% for most datasets, with DeepPlant showing a slight advantage in *S. miltiorrhiza*, *R. communis*, *O. sativa*, and *B. vulgaris*. In *S. miltiorrhiza* and *O. sativa*, DeepPlant surpassed Dorado by over 1%. At 15× depth, DeepPlant consistently led across all species, with more than a 1% advantage in *S. miltiorrhiza*, *O. sativa*, and *B. vulgaris*. At 30× depth, DeepPlant's lead increased, showing a 2.51%, 3.28%, and 2.83% improvement in *S. miltiorrhiza*, *O. sativa*, and *A. thaliana*, respectively, and 1.31% in *B. vulgaris*.

For CpG methylation, DeepPlant performed comparably to Dorado, with differences remaining within 1% across most species. DeepPlant slightly outperformed Dorado in *V. vinifera*, *R. communis*, *S. lycopersicum*, and *A. thaliana*, while *S. tuberosum* and *C. sinensis* saw minor gains for Dorado. In *O. sativa*, DeepPlant held a notable advantage, leading by 2.84% at 5× depth and 1.67% at 30× depth compared to Dorado. Compared to Rockfish, DeepPlant exhibits a slight advantage across all datasets except for *C. sinensis*, with the differences being within 1%. Full quantitative results are provided in Supplementary Data 6.

Supplementary Note 2. Single-molecule performance evaluation of DeepPlant for CpG and CHG methylation detection

In single-molecule evaluations for CHG methylation, DeepPlant outperformed Dorado across all species. DeepPlant's F1-scores exceeded Dorado by over 5% in *S. tuberosum*, *S. miltiorrhiza*, *R. communis*, *O. sativa*, and *B. vulgaris*, with a 9.3% lead in *S. miltiorrhiza*. In *V. vinifera* and *A. thaliana*, DeepPlant led by 4.50% and 2.78%, respectively. Additionally, DeepPlant showed a 1.49%-5.70% improvement in AUROC and a 1.80%-5.56% increase in Average Precision (AP), with significant gains in *S. tuberosum*, *S. miltiorrhiza*, and *R. communis*. In eight of the nine species, excluding *A. thaliana*, DeepPlant achieved AUROC and AP scores exceeding 0.98.

For CpG methylation, DeepPlant consistently outperformed Dorado across all nine species, with F1-scores 1.04%-7.45% higher, particularly in *S. tuberosum*, *S. miltiorrhiza*, and *O. sativa*. DeepPlant also improved AUROC by 1.11%-3.64% and AP by 1.27%-3.46%, with *S. tuberosum* and *O. sativa* showing increases of more than 3%. DeepPlant achieved AUROC and AP scores above 0.98 in seven species, demonstrating superior accuracy and recall across all species compared to Dorado. Compared to Rockfish, DeepPlant exhibits a slight advantage in metrics such as AUC, AP, F1-score, Accuracy, and Recall across most datasets except for *C. sinensis*. On the other hand, Rockfish shows a marginal lead in Precision, Specificity across most datasets, indicating a tendency to classify samples as negative. Full single-molecule evaluation results are available in Supplementary Data 6.
