# Supplementary material for: Accurate cross-species 5mC detection for Oxford Nanopore sequencing in plants with DeepPlant
Source: Nat Commun. 2025 Apr 4;16:3227. doi: 10.1038/s41467-025-58576-x (PMC11971355; doi:10.1038/s41467-025-58576-x)
Supplement: Supplementary file 2 — Description of Additional Supplementary Files [file 41467_2025_58576_MOESM2_ESM.pdf]

Description of Additional Supplementary Files

File Name: Supplementary Data 1

Description: **Reanalyzed public BS-seq data of 10 plant species.**

File Name: Supplementary Data 2

Description: **BS-seq and Nanopore sequencing information and the number of samples for site-level evaluation.**

File Name: Supplementary Data 3

Description: **The ablation experiments on the triple-encoder, the models were evaluated on chromosome NC_003070.9 of A. thaliana and chromosome NC_029256.1 of O. sativa.**

File Name: Supplementary Data 4

Description: **Hyperparameters of DeepPlant models.**

File Name: Supplementary Data 5

Description: **Performance of DeepPlant CHH motif 9-mer Bi-LSTM model on methylation frequency quantification.** Nanopore datasets were randomly down-sampled to 5-30× if possible. Whole-genome methylation frequency correlation with BS-seq were carried out for each down-sampled dataset.

File Name: Supplementary Data 6

Description: **Comparison of methylation frequency correlation with BS-seq and single-molecule methylation detection performance between DeepPlant and Dorado on CpG and CHG motifs.** 5-30× denote the Pearson correlation (r) between whole-genome methylation frequencies of nanopore data at corresponding down-sampled depth with corresponding BS-seq datasets. Single-molecule evaluation was conducted by extracting 200,000 samples per iteration, with the process repeated three times. The single-molecule evaluation results are presented in the form of mean ± standard deviation.

File Name: Supplementary Data 7

Description: **Performance of DeepPlant Bi-LSTM model and Dorado at single-molecule level on CHH motifs under imbalanced conditions.** The models were evaluated at unsampled single chromosome or whole genome data, with bisulfite analysis showing methylation frequencies of **100% for positive samples** and **0% for negative samples** and the sequencing coverage higher than 5×.

File Name: Supplementary Data 8

Description: **Performance of DeepPlant Bi-LSTM model and Dorado at single-molecule level on CHH motifs.** Single-molecule evaluation was conducted by extracting 200,000 samples per iteration, with the process repeated three times. The evaluation results are presented in the form of **mean ± standard deviation**.

File Name: Supplementary Data 9

Description: **Average nanopore sequencing depth of centromeres regions in O. sativa.**

File Name: Supplementary Data 10

Description: **Execution time and memory usage for each ONT-base tool (R10.4.1) on the Arabidopsis**

thaliana R10.4.1 dataset (1,596,813 reads). Both tools call all 5mC contexts including CHH, CHG and CpG. The benchmarking was performed on a computer with an AMD CPU (Eng Sample: 100-000000894-04) and an NVIDIA L40S GPU.
