# Supplementary material for: Accurate cross-species 5mC detection for Oxford Nanopore sequencing in plants with DeepPlant
Source: Nat Commun. 2025 Apr 4;16:3227. doi: 10.1038/s41467-025-58576-x (PMC11971355; doi:10.1038/s41467-025-58576-x)
Supplement: Supplementary file 13 — Report Summary [file 41467_2025_58576_MOESM13_ESM.pdf]

nature portfolio

Corresponding author(s): Chuanle Xiao

Last updated by author(s): Mar 21, 2025

Reporting Summary

Nature Portfolio wishes to improve the reproducibility of the work that we publish. This form provides structure for consistency and transparency in reporting. For further information on Nature Portfolio policies, see our Editorial Policies and the Editorial Policy Checklist.

Statistics

For all statistical analyses, confirm that the following items are present in the figure legend, table legend, main text, or Methods section.

n/a	Confirmed	
☐	☒	The exact sample size (*n*) for each experimental group/condition, given as a discrete number and unit of measurement
☐	☒	A statement on whether measurements were taken from distinct samples or whether the same sample was measured repeatedly
☐	☒	The statistical test(s) used AND whether they are one- or two-sided<br>*Only common tests should be described solely by name; describe more complex techniques in the Methods section.*
☒	☐	A description of all covariates tested
☐	☒	A description of any assumptions or corrections, such as tests of normality and adjustment for multiple comparisons
☐	☒	A full description of the statistical parameters including central tendency (e.g. means) or other basic estimates (e.g. regression coefficient) AND variation (e.g. standard deviation) or associated estimates of uncertainty (e.g. confidence intervals)
☐	☒	For null hypothesis testing, the test statistic (e.g. *F*, *t*, *r*) with confidence intervals, effect sizes, degrees of freedom and *P* value noted<br>*Give P values as exact values whenever suitable.*
☒	☐	For Bayesian analysis, information on the choice of priors and Markov chain Monte Carlo settings
☐	☒	For hierarchical and complex designs, identification of the appropriate level for tests and full reporting of outcomes
☐	☒	Estimates of effect sizes (e.g. Cohen's *d*, Pearson's *r*), indicating how they were calculated

Our web collection on statistics for biologists contains articles on many of the points above.

Software and code

Policy information about availability of computer code

Data collection	The BS-seq and Nanopore sequencing data of A. thaliana, O. sativa and B. vulgaris are downloaded from BIG Data Center, Beijing Institute of Genomics under Project Accession No. PRJCA023349. The BS-seq and Nanopore sequencing data of HG002 are downloaded from Oxford Nanopore's EPI2ME platform (https://labs.epi2me.io/gm24385-5mc). And other data were generated by our sequencing experiments.
Data analysis	This study utilized open software and packages, which are listed below: Dorado (0.8.1); minimap2 (2.27-r1193); PyTorch (2.0.1). 5mC modifications were called with the model version dna_r10.4.1_e8.2_400bps_hac@v5.0.0. And custom code and scripts generated within this project are available at https://github.com/xiaochuanle/DeepPlant.

For manuscripts utilizing custom algorithms or software that are central to the research but not yet described in published literature, software must be made available to editors and reviewers. We strongly encourage code deposition in a community repository (e.g. GitHub). See the Nature Portfolio guidelines for submitting code & software for further information.

Data

Policy information about availability of data

All manuscripts must include a data availability statement. This statement should provide the following information, where applicable:

- Accession codes, unique identifiers, or web links for publicly available datasets
- A description of any restrictions on data availability
- For clinical datasets or third party data, please ensure that the statement adheres to our policy

All sequencing data generated in this study (BS-seq and nanopore sequencing data of S. miltiorrhiza, S. tuberosum, R. communis, C. sinensis, S. lycopersicum, and V. vinifera; BS-seq data of G. max, P. patens and M. polymorpha) and the assembled reference genome of V. vinifera have been deposited in the Genome Sequence Archive in BIG Data Center, Beijing Institute of Genomics (BIG, http://gsa.big.ac.cn) under Project Accession No. PRJCA030666. The BS-seq and Nanopore sequencing data of A. thaliana, O. sativa, and B. vulgaris are available at BIG under Project Accession No. PRJCA023349. The reference genomes of S. miltiorrhiza (GCF_028751815.1), S. tuberosum (GCF_000226075.1), R. communis (GCF_019578655.1), C. sinensis (GCF_022201045.2), A. thaliana (GCF_000001735.4), O. sativa (GCF_034140825.1), S. lycopersicum (GCA_915070445.1), and B. vulgaris (GCF_026745355.1) were downloaded from NCBI. The genome assembly and annotations for the T2T-NIP of O. sativa were accessed from RiceSuperPIRdb (http://www.ricesuperpir.com/web/nip). For Figure 3c,d,e, Supplementary Figure 3a-c, and Supplementary Figure 6a-c, the source data files are large and provided at https://doi.org/10.5281/zenodo.15062213. The source data of all the rest figure panels are provided with this paper.

Research involving human participants, their data, or biological material

Policy information about studies with human participants or human data. See also policy information about sex, gender (identity/presentation), and sexual orientation and race, ethnicity and racism.

Reporting on sex and gender	N/A
Reporting on race, ethnicity, or other socially relevant groupings	N/A
Population characteristics	N/A
Recruitment	N/A
Ethics oversight	N/A

Note that full information on the approval of the study protocol must also be provided in the manuscript.

Field-specific reporting

Please select the one below that is the best fit for your research. If you are not sure, read the appropriate sections before making your selection.

☒ Life sciences ☐ Behavioural & social sciences ☐ Ecological, evolutionary & environmental sciences

For a reference copy of the document with all sections, see nature.com/documents/nr-reporting-summary-flat.pdf

Life sciences study design

All studies must disclose on these points even when the disclosure is negative.

Sample size	Data from three species were applied in model training, and data from another six plant species were sequenced and subjected to testing as biological replicates. Based on the characteristics of Nanopore sequencing technology, a sequencing depth of 30× is considered sufficient to reach saturation. Following this criterion, we determined the appropriate sequencing depth.
Data exclusions	No data was excluded.
Replication	The performance evaluation of the DeepPlant model was evaluated across datasets collected from six different plant species.
Randomization	Down-sampling was random wherever performed in this study.
Blinding	Blinding is not relevant to this study.

Reporting for specific materials, systems and methods

We require information from authors about some types of materials, experimental systems and methods used in many studies. Here, indicate whether each material, system or method listed is relevant to your study. If you are not sure if a list item applies to your research, read the appropriate section before selecting a response.

Materials & experimental systems

n/a	Involved in the study	
☒	☐	Antibodies
☒	☐	Eukaryotic cell lines
☒	☐	Palaeontology and archaeology
☒	☐	Animals and other organisms
☒	☐	Clinical data
☒	☐	Dual use research of concern
☐	☒	Plants

Methods

n/a	Involved in the study	
☒	☐	ChIP-seq
☒	☐	Flow cytometry
☒	☐	MRI-based neuroimaging

Dual use research of concern

Policy information about dual use research of concern

Hazards

Could the accidental, deliberate or reckless misuse of agents or technologies generated in the work, or the application of information presented in the manuscript, pose a threat to:

No	Yes	
☒	☐	Public health
☒	☐	National security
☒	☐	Crops and/or livestock
☒	☐	Ecosystems
☒	☐	Any other significant area

Experiments of concern

Does the work involve any of these experiments of concern:

No	Yes	
☒	☐	Demonstrate how to render a vaccine ineffective
☒	☐	Confer resistance to therapeutically useful antibiotics or antiviral agents
☒	☐	Enhance the virulence of a pathogen or render a nonpathogen virulent
☒	☐	Increase transmissibility of a pathogen
☒	☐	Alter the host range of a pathogen
☒	☐	Enable evasion of diagnostic/detection modalities
☒	☐	Enable the weaponization of a biological agent or toxin
☒	☐	Any other potentially harmful combination of experiments and agents

Plants

Seed stocks

Callus cultures were established from undeveloped ovules of C. sinensis cv. 'Liucheng' and leaf discs of Vitis vinifera var. 'Baiti'. Fresh roots of wild S. miltiorrhiza were collected in March 2024 from Song County, Henan, China. Embryos of R. communis were separated from fresh seeds of wild plants collected in March 2024 from Maoming, Guangdong, China. Outer pericarps of S. lycopersicum (cultivar DRK0568) were dissected for DNA extraction. A tuber from the S. tuberosum variety A9, with the epidermis removed, was cut into 0.5 cm cubes.

Novel plant genotypes

N/A

Authentication

The taxonomic species of the plants were verified through being mapped to their respective reference genome.
